# Complete regression of branching vascular network in polypoidal choroidal vasculopathy by ranibizumab and photodynamic therapy, two case reports

**DOI:** 10.1186/s12886-018-0952-6

**Published:** 2018-11-03

**Authors:** Yasuhiro Iesato, Masaaki Tanaka, Masako Murata, Junya Kitahara, Takao Hirano, Taihei Kurenuma, Noriko Yoshida, Toshinori Murata

**Affiliations:** 1 Department of Ophthalmology, School of Medicine, Shinshu University, 3-1-1 Asahi, Matsumoto, Nagano, 390-8621 Japan; 2 Department of Ophthalmology, Matsumoto Medical Center, Narional Hospital Organaization, 2-20-30 Murai-Minami, Matsumoto, Nagano, 390-8621 Japan

**Keywords:** Polypoidal choroidal vasculopathy, Branching vascular network, Polypoidal lesions, Ranibizumab, Photodynamic therapy, Optical coherence tomography angiography

## Abstract

**Background:**

Polypoidal choroidal vasculopathy (PCV) consists of polyps that potentially cause massive subretinal hemorrhage and their branching vascular network (BVN) of feeder vessels. Although conventional indocyanine green angiography (IA) has shown anti-vascular endothelial growth factor (VEGF) agents and/or photodynamic therapy (PDT) to successfully induce polyp closure, the BVN appears resistant to these therapies and serves as the origin of recurrent active polyps. Recently introduced optical coherence tomography angiography (OCT-A) enables more frequent angiographic evaluation of polyps and the BVN than does conventional IA since it does not require intravenous fluorescent dye injection and is thus considered non-invasive.

**Case presentation:**

Case 1. A 70-year-old male with PCV in his left eye suffered from vision deterioration (20/40) due to persistent subretinal fluid despite 42 intravitreal injections of ranibizumab (IVRs) over 5 years and 7 months. PDT was performed as an adjunct therapy 3 days after the 43rd IVR. IA at 3 months after PDT showed successful polyp closure but persisting BVN. However, more frequent evaluation with OCT-A starting at 1 week after PDT demonstrated complete regression of both the BVN and polyp. OCT-A at every subsequent outpatient visit depicted gradual re-perfusion of the BVN and the restoration of most of its original network at 3 months, which was compatible with IA findings. Neither OCTA nor IA revealed polyp recurrence at 3 months.

Case 2. A 65-year-old female suffering from left vision deterioration due to PCV underwent 5 intravitreal injections of aflibercept. Since her subretinal fluid persisted, the treatment was switched to a combination of IVR and PDT. OCT-A revealed marked regression of the BVN and polyp at 2 weeks, but the BVN had regained its original shape at 2 months without any sign of polyp recurrence.

**Conclusions:**

Differently from previous observations obtained by IA alone, more frequent non-invasive OCT-A examination revealed complete but transient regression of the BVN just after combination therapy with IVR and PDT.

## Background

Polypoidal choroidal vasculopathy (PCV) is a subtype of neovascular age-related macular degeneration (AMD) that accounts for 22.3–61.6% of neovascular AMD patients in Asia [1, 2]. PCV consists of a branching vascular network (BVN) and its characteristic terminal polyps, both of which are located between the retinal pigment epithelium (RPE) and Bruch’s membrane [3–5]. The rupture of polyps in PCV can lead to massive subretinal hemorrhage and cause sudden and severe vision deterioration [6]. Another devastating nature of polyps even after successful treatment of PCV is their high recurrence rate and eventual severe vision loss [2]. Many treatment modalities for PCV have been evaluated in terms of their polyp closure rate. Indocyamine green angiography (IA) has been essential for accurately monitoring the regression of polyps and BVN since it employs a longer wavelength than does fluorescein angiography (FA) to provide more fluorescence through the melanin pigments of the RPE and more clearly depict the polyps and BVN underneath.

The major treatments for PCV are anti-vascular endothelial growth factor (VEGF) agents, photodynamic therapy (PDT), and a combination of both [1, 2]. All 3 treatment modalities could provide relatively high polyp closure rate, while BVN persists in follow-up FA/IA usually performed 3 months or later after the initiation of treatments. [2, 7–13]

Optical coherence tomography angiography (OCT-A) is a modern technique that depicts retinal and choroidal vessels by detecting flow signals. Consequently, it is considered a non-invasive form of angiography not requiring intravenous injection of fluorescent dye. Inoue et al. reported that en face images from OCT-A provided anatomical information about the BVN that was comparable to that from IA. Polyps were less clearly depicted in the en face OCT-A images than on IA but were clearly defined in cross-sectional OCT-A images with flow signals [14, 15]. Since OCT-A is non-invasive and requires only a few seconds for acquiring retino-choroidal vascular images, more frequent and intensive longitudinal follow-up of the BVN and polyps is now possible. Indeed, anaphylactic shock induced by IA is extremely rare but possible [16], resulting in more conservative use by clinicians.

The present study analyzed the early changes of BVN and polyps that had been resistant to multiple anti-VEGF agents following combination therapy of IVR and PDT. From as early as 1 week after treatment, OCT-A was performed at monthly visits and the findings at 3 months were compared with those of IA.

## Case presentation

### Case 1

**Fig. 1:**
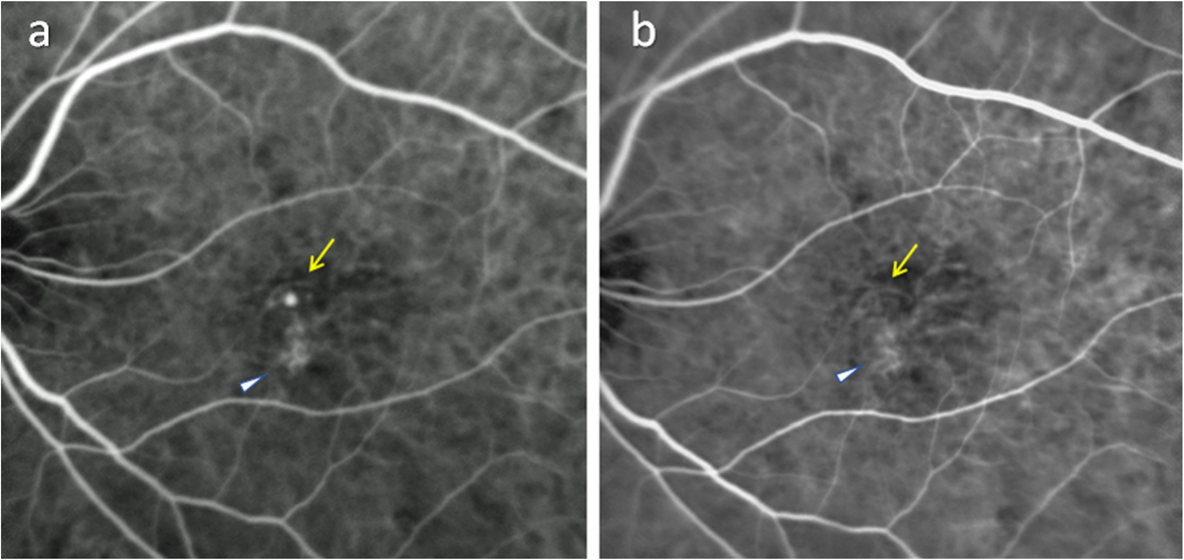
Indocyaning green angiography before and after combination treatment of intravitreal ranibizumab injection and photodynamic therapy. Indocyanine green angiography just before combination therapy with the branching vascular network (arrowhead) and a polyp (yellow arrow) of polypoidal choroidal vasculopathy evident (**a**). Three months after photodynamic therapy, complete regression of the polyp (yellow arrow) is confirmed while the branching vascular network persists (arrowhead) (**b**)

A 68-year-old man with left vision deterioration presented to our outpatient clinic in August 2011. His best corrected visual acuity (BCVA) was 20/20 in the right eye and 20/40 in the left eye. Anterior segment examination was unremarkable. Dilated fundus examination revealed red-orange lesions in the macula associated with RPE damage. IA (Spectralis HRA, Heidelberg Engineering, Heidelberg, Germany) revealed a BVN and polyps. OCT (Carl Zeiss Meditec, Dublin, CA) disclosed significant subretinal fluid with RPE detachment. Based on these findings, a diagnosis of PCV was made and IVRs were initiated in a pro re nata (PRN) regimen. After 42 anti-VEGF injections, 7 initial IVRs and subsequent 35 IVAs, over 5 years and 7 months, his left vision remained at 20/40, but OCT showed persistent subretinal fluid and RPE detachment. As IA demonstrated a polyp associated with the BVN (Fig. 1a), his treatment strategy was switched to combination IVR and PDT according to the EVEREST II study [17]. IA 3 months subsequent to PDT confirmed complete regression of polyp, with the BVN persisting without apparent regression (Fig. 1b).

**Fig. 2:**
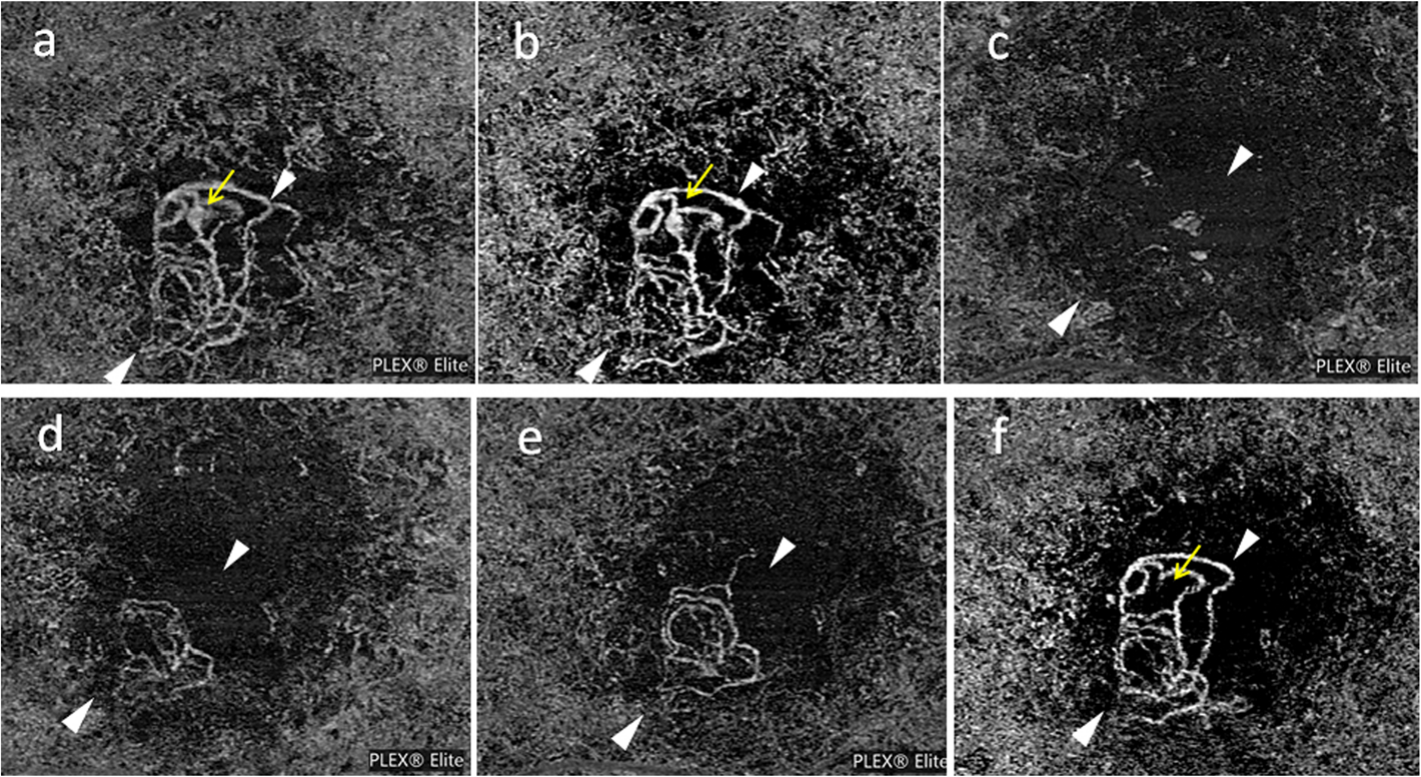
Longitudinal observation of branching vascular network and polyp using optical coherence tomography angiography. Nine months before combination therapy of photodynamic therapy (PDT) and intravitreal injection of ranibizumab (IVR), en face optical coherence tomography angiography (OCT-A) reveals the branching vascular network (BVN; between arrowheads) and a polyp (yellow arrow). This eye had previously undergone 33 IVRs (**a**). After 9 additional IVRs, the BVN and polyp show no apparent regression (**b**). One week after combination IVR (43rd) and PDT, OCT-A confirms complete regression of the BVN and polyp (**c**). BVN shows gradual reperfusion at 1 month (**d**) and 2 months (**e**). At 3 months, the BVN has restored most of its original network (between arrowheads) while the polyp remains absent (yellow arrow) (**f**)

In this patient, the first OCT-A (PLEX Elite 9000; Carl Zeiss Meditec, Dublin, CA) image (Fig. 2a) was acquired 9 months prior to combination therapy and disclosed the BVN and polyp in the corresponding locations as indicated by IA (Fig. 1a). Before PDT incorporation, the eye received 9 additional IVRs, but OCT-A after each injection showed no apparent changes in the BVN or polyp (Fig. 2b). Based on these findings, the treatment strategy was switched to combined therapy of IVR and PDT. Standard full-fluence PDT was performed 3 days after the 43rd IVR, followed by PRN-IVR for subretinal or intraretinal fluid. In full fluence PDT, patients were infused with verteporfin (6 mg/m^2^). Fifteen minutes after the start of infusion, PDT at standard fluence (light dose, 50 J/cm^2^; dose rate, 600 mW/cm^2^; wavelength, 689 nm) was applied to the eye for 83 s. The laser spot size was derived by adding 1000 mm to the greatest linear dimension. Thus, both BVN and polyps were included in the verteporfin PDT treatment area. The effects of combination therapy on the BVN and polyp were investigated by frequent OCT-A performed at every visit to our outpatient clinic. As early as 1 week after PDT, OCT-A revealed complete regression of both the BVN and polyp (Fig. 2c). Right vision improved from 20/40 to 20/30. The BVN showed gradual reperfusion at 1 month (Fig. 2d) and 2 months (Fig. 2e), and had virtually restored its original appearance at 3 months while the polyp remained closed (Fig. 2f).

**Fig. 3:**
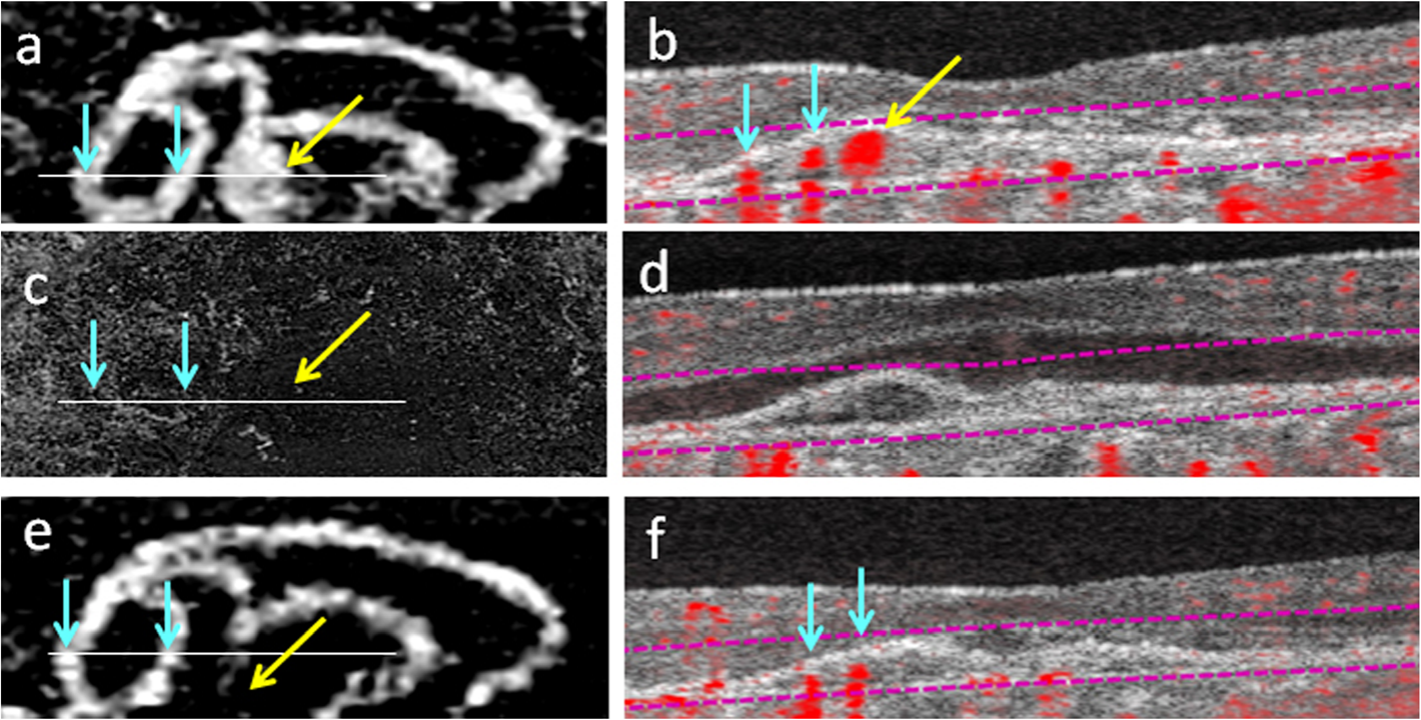
En face optical coherence tomography angiography and corresponding cross-sectional optical coherence tomography angiography with flow signals of polypoidal choroidal vasculopathy in case 1. Just before combination therapy of intravitreal injection of ranibizumab (IVR) and photodynamic therapy (PDT), en face optical coherence tomography angiography (OCT-A) shows the branching vascular network (BVN, blue arrows) and a polyp (yellow arrow) (**a**). Cross-sectional OCT-A on the plane of the white line in (**a**) shows pigment epithelium detachment (PED) containing a total of 3 flow signals that correspond to the BVN (2 blue arrows) and polyp (yellow arrow) (**b**). OCT-A at 1 week after PDT shows complete regression of both the BVN and polyp (**c**), which can be confirmed in cross-sectional OCT-A that does not show any flow signals in the mildly flattened PED (**d**). Three months after PDT, the BVN has restored most of its original network (blue arrows) observed in (**a**) while the polyp remains absent (yellow arrow) (**e**). In cross-sectional OCT-A at 3 months, 2 flow signals that correspond to the restored branches of the BVN are present (blue arrows) and flow signals for the polyp are missing (**f**)

We confirmed the changes observed in en face OCT-A images using corresponding OCT-A cross-sectional scans. The en face OCT-A data acquired just before PDT showed the polyp as an aneurysm-like dilatation of the BVN (Fig. 3a). A cross-sectional OCT-A (Fig. 3b) image of the PED (the space between Bruch’s membrane and the RPE layer) contained a toal of 3 major flow signals corresponding to the polyp and 2 branches of the BVN, respectively. One week after PDT when en face OCT-A demonstrated complete regression of the BVN and polyp (Fig. 3c), the residual space between Bruch’s membrane and the RPE layer on cross-sectional OCT-A suggested that the BVN was occluded but the vessel structures were still preserved (Fig. 3d). The en face OCT-A taken 3 months after PDT demonstrated a very similar BVN to that beforehand except for the absence of the polyp (Fig. 3e). Cross-sectional OCT-A confirmed that the flow signals corresponding to branches of the BVN were indeed restored but the polyp remained absent (Fig. 3f). At 3 months after PDT, IA also visualized the BVN, which showed a near-identical appearance as that before combination therapy, along with absence of the polyp (Fig. 1b).

### Case 2

**Fig. 4:**
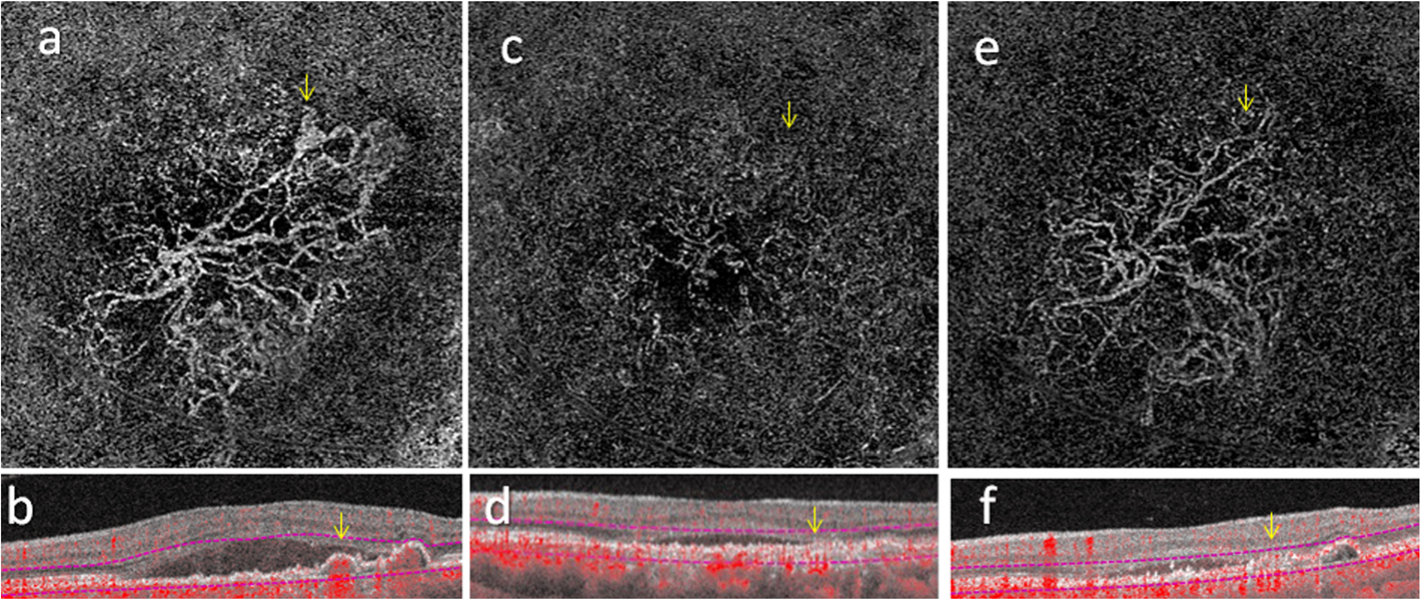
En face optical coherence tomography angiography and corresponding cross-sectional optical coherence tomography angiography with flow signals of polypoidal choroidal vasculopathy in case 2. Prior to combination therapy of intravitreal injection of ranibizumab (IVR) and photodynamic therapy (PDT), en face optical coherence tomography angiography (OCT-A) shows the branching vascular network (BVN) and a polyp (yellow arrow) (**a**). A corresponding cross-sectional OCT-A demonstrates the pigment epithelium detachment (PED) to contain flow signals (red dots indicated by yellow arrow) at the position of the polyp (**b**). OCT-A 2 weeks after PDT shows marked regression of both the BVN and polyp (yellow arrow) (**c**), which can be confirmed in cross-sectional OCT-A depicting a flattened PED devoid of flow signals (**d**). Two months after PDT, the BVN has restored most of its original network observed in (**a**) while the polyp remains absent (yellow arrow) (**e**). In cross-sectional OCT-A at 2 months, the PED shows mild restoration of height but flow signals are still minimal (**f**)

A 65-year-old woman presented with left vision deterioration in July 2017. Her BCVA was 20/20 in the right eye and 20/22 in the left eye. Anterior segment examination was unremarkable. Dilated fundus examination revealed orange nodular lesions in the macula associated with exudative changes. IA uncovered a BVN and polyps. OCT disclosed subretinal fluid with PED. Based on these findings, a diagnosis of PCV was made and she began IVA treatment. After 5 consecutive monthly IVAs, OCT showed persistent subretinal fluid and PED, and so we switched her to combination therapy of IVR and PDT according to the EVEREST II study [17]. En face OCT-A just before PDT revealed a fan-shaped BVN and a polyp (Fig. 4a), and corresponding cross-sectional OCT-A at the plane of the polyp demonstrated dome-shaped PED containing flow signals (Fig. 4b). Two weeks after PDT, OCT-A showed a trace of the BVN and complete regression of the polyp (Fig. 4c). Cross sectional OCT-A revealed flattened PED that was devoid of flow signals (Fig. 4d). Left vision improved from 20/60 to 20/20. En face OCT-A at 2 months indicated that the BVN had largely restored its original shape but the polyp was still absent (Fig. 4e). In corresponding cross-sectional OCT-A, the PED had mildly restored its height, with no apparent restoration of flow signals (Fig. 4f). The patient was lost to follow-up after her 2-month visit.

## Discussion and conclusions

In PCV, exudative changes including subretinal hemorrhage are usually derived from active polyps [6, 7, 18–21]. Consequently, polyp closure is important for its successful treatment. Anti-VEGF monotherapy is currently the first line treatment for AMD, including PCV [1, 19]. However, anti-VEGF therapy using ranibizumab or bevacizumab has a limited effect on PCV, with polyp regression rates ranging from 26 to 33% over a period of 1 year [12, 13]. Moreover, BVN size increased in most cases [22, 23]. Intravitreal injection of aflibercept (IVA) has recently been reported to be effective for PCV, producing a 51.8–72.5% polyp regression rate in cohorts that included patients responding poorly to ranibizumab, although the BVN persisted in all eyes [7, 18–21].

PDT could achieve a 95% polyp regression rate over 1 year, but BVN regression remained minimal. Persistent BVN often serves as the origin of the recurring or newly developed polyps associated with subretinal hemorrhage and pigment epithelium tear over years of follow-up [2, 7–11]. Consequently, the long-term visual outcome of PDT is negative despite a high polyp closure rate.

In the EVEREST II study [17], combination therapy of intravitreal ranibizumab injection (IVR) and PDT achieved superior vision outcomes than did IVR monotherapy: higher polyp closure rates were obtained with less frequent IVRs and complete polyp regression rates at months 3, 6, and 12 were consistently higher for combination therapy (71.4%, 71.3%, and 69.7%, respectively) than for ranibizumab monotherapy (23.3%, 28.0%, and 33.8%, respectively). However, not even combination therapy could induce complete regression of the BVN, leaving a risk of recurrent active polyp development [8].

There have been detailed reports about early FA/IA changes of choroidal neovascularization (CNV), which showed regression in 5 h and became inapparent 1 day after PDT in both FA/IA. At 3 months, however, the CNV size was consistently larger than at baseline [24, 25]. On the other hand, there have been no reports on the early morphological changes of the BVN and polyps in PCV soon after intravitreal injection of anti-VEGF drugs and/or PDT.

We herein present sequential early OCT-A evidence on how combination therapy of IVR and PDT induces transient, but complete, regression of BVN and polyps that had persisted after multiple intravitreal injections of anti-VEGF agents. OCT-A is non-invasive since it does not require intravenous dye injection to visualize retino[24]-choroidal vessels, which enabled frequent angiographic examination as early as 1 week after combination therapy. In contrast, the first IA was performed 3 months after treatment in many earlier studies to minimize the potential risk of anaphylactic shock.

OCT-A examination of the retino-choroidal circulation was possible at every outpatient clinic visit. In case 1, 42 anti-VEGF injections had been given to maintain adequate vision. After we started to use OCT-A in our outpatient clinic,9 IVRs were performed but OCT-A revealed no signs of BVN or polyp regression. As subretinal fluid persisted, we performed additional PDT 3 days after the 43rd IVR based on the EVEREST II study [17]. Surprisingly, OCT-A revealed complete regression of the BVN and polyp 1 week later. BVN gradually displayed reperfusion and restored trunk vessels in OCT-A at 3 months, almost perfectly resembling the pre-PDT findings. Conventional IA at that time confirmed the hyperfluorescence indicative of the BVN. We previously evaluated the treatment of PCV by IA only and generally believed that anti-VEGF agents, PDT, or their combination had no effect on the BVN while inducing polyp closure. However, more frequent OCT-A examination indicated that PDT induced transient complete regression of the BVN. Early cross-sectional OCT-A depicted both PED flattening and loss of flow signals, suggesting collapse of the BVN lumen and associated polyp. Although the BVN had restored its original network in 3 months, neither OCT-A nor IA showed the recurrence of polyps.

In case 2, OCT-A was performed 2 weeks after PDT. Regression of the BVN was remarkable but traces of BVN trunk vessels could already be distinguished. Cross-sectional OCT-A showed the disappearance of flow signals without PER flattening. These findings indicated that blood flow was reduced in the BVN but that vessel lumens were still present.

The above cases show OCT-A to be a useful tool for investigating the effects of anti-VEGF agents and PDT in the PCV treatment. Through OCT-A, we were able to characterize the changes in BVN and polyp status much more frequently than by IA alone.

Differently from previous observations by IA, PDT in conjunction with anti-VEGF agents were found by OCT-A to induce complete regression of the BVN, which then reappeared within 3 months. Since polyps are usually formed at the terminal portions of the BVN, such early-stage regression may explain the superior effects of combination therapy in polyp closure and visual outcomes in the EVEREST II study.

The limitations of this study are very low number of observed patients and their short follow up period. This study included only two PCV patients and one of them failed to visit our outpatient clinic on month 3, when the findings of OCT-A and IA were compared. Larger prospective trials including OCT-A monitoring are warranted to better characterize the nature of BVN and PCV, and merits of combination IVR and PDT treatment.

## Abbriviations

AMD: age-related macular degeneration
BVN: branching vascular network
IA: indocyanine green angiography
IVA: intravitreal injections of aflibercept
IVRs: intravitreal injections of ranibizumab
OCT-A: optical coherence tomography angiographic
PCV: Polypoidal choroidal vasculopathy
PDT: photodynamic therapy
RPE: retinal pigment epithelium
VEGF: vascular endothelial growth factor
